# Patient prioritization and management during the COVID-19 pandemic

**DOI:** 10.1177/15910199211035302

**Published:** 2021-10-20

**Authors:** Mohamed Aggour, Cuong Tran Chi, Jens Fiehler

**Affiliations:** 1 Department of Interventional Neuroradiology, 112001 The Royal London Hospital, UK; 2 Stroke International Services (S.I.S) General Hospital, Vietnam; 3 Department of Diagnostic and Interventional Neuroradiology, UMC Hamburg-Eppendorf, Germany

**Keywords:** SARS-CoV-2, COVID-19, patient prioritization

## Abstract

The coronavirus disease 2019 (COVID-19) pandemic affected the healthcare system in a major way generally. Healthcare re-organization of resources and manpower, establishing management protocols and specific patients’ pathways are all evolving with the continuously changing situation. Neuro-vascular management and its re-organization are part of these global measures to cope with this pandemic in a way to establish less risky patients’ pathways, help in patients’ triage, protecting the staff by introducing training and applying safety measures and to manage neuro-vascular emergencies and elective activity. We here describe the situation of the pandemic affecting neuro-vascular interventions and propose our recommendations for patients’ triage, resources management and organization, remote solutions and preparations for any future waves.

The novel severe acute respiratory syndrome coronavirus 2 (SARS-CoV-2), the causative agent of coronavirus disease 2019 (COVID-19), was first identified in Wuhan, China.^1^ After few months of its first emergence, COVID-19 was declared a global pandemic by the World Health Organization on 11 March 2020 and the whole world since then is experiencing a new era. This pandemic had enormous effects on every aspect of life and exerted a huge burden on healthcare systems. In order to cope with this huge influx of patients and drainage of hospital resources, medical bodies had to set rules and recommendations to organize the given care. All this was done and updated periodically while at the same time trying to study and understand more the disease itself and its evolution.

## COVID-19 and neurovascular activity, management and manifestations

There were considerable variations in the management of neurointerventions. Several surveys have showed the significant alteration in neuroendovascular management and activity.^2–6^ In a global survey, the vast majority of the 475 participants (96%) reported being able to provide emergency services while a decrease in emergency procedures was observed in 69% of the centres. Only 4% reported an increase in emergency cases. Two-thirds of the participants had abandoned neurointerventions in non-emergent situations.

Many European stroke physicians reported changes in their working situation, either related to new activities or modifications in the work schedules, some described extended working hours due to a lack of personnel and others reported the need to contribute to new tasks outside stroke care. Not all stroke patients were receiving the usual care in most of the centres, and some centres avoided admitting patients whenever possible and others described lack of beds for stroke patients.^2^

In a French study, 844 (55.8%) patients were treated in 2019 and 668 (44.2%) in 2020, representing an overall 20.9% drop in patients receiving mechanical thrombectomy (MT) during the pandemic period.^7^ This constituted a positive correlation after March 15th, between the total number of hospitalizations for COVID-19 and the number of MT cases in France. During the pandemic period, there was a significant increase in delays between imaging and groin puncture, overall and in patients transferred for MT after imaging. This decrease might be the consequence of the struggle to access transfer resources by the primary stroke centres (PSC) within the therapeutic window, physicians being strict to treat only patients within the guidelines criteria or patient's and family fear to send their relatives to hospitals.^5^ Moreover, the in-hospital triaging of patient of unknown COVID-19 status might be another contributing factor leading to increases of door-to-groin times, particularly if the patients require general anaesthesia, as many COVID + stroke patients do require.

In Germany, the number of patients hospitalized for stroke or transient ischemic attack between declined by 7% for stroke and by 13% with the lowest levels in April and May 2020.^7^ Public awareness is an important issue to be managed during pandemic times as it may affect the delay between stroke onset to hospital arriving time and also may affect the number of patients presenting with stroke.^2^

A survey from the United States reported reduced MT procedures with 32% reporting a greater than 50% reduction in thrombectomy volumes. A dramatic reduction of more than 50% in overall neurointerventional procedural volume was reported by 68% of physicians. Increased door-to-puncture times were reported by 79%. The 66% of respondents reported increased career stress, 56% increased personal life/family stress and 35% increased career burnout.^3^

Based on data of Big Data Observatory Platform for Stroke of China, the total number of thrombolysis and thrombectomy cases dropped by 27% and 25%, respectively, in February 2020 as compared with February 2019. The capacity for stroke care was reduced in the majority of the hospitals. Most of the stroke centres stopped or reduced their efforts in stroke education for the public. Hospital admissions related to stroke dropped by around 40%; thrombolysis and thrombectomy cases dropped by around 25%. The authors concluded that many factors contributed to the reduced admissions and pre-hospital delays; lack of stroke knowledge and proper transportation were significant limiting factors. Patients’ not coming to the hospital for fear of virus infection was another likely key factor.^6^

Based on Vietnamese Ministry of Health statistics, in 1168 COVID-19 infectious cases, 1057 (90.5%) patients have been cured and have had only 35 (3%) have died. These positive numbers manifest the efforts of the government and the healthcare system in particular. Prevention from epidemic areas begins with immigration control and early infectious individual recognition and treatment. In a survey from one stroke centre in Mekong Delta/Southeast Vietnam – S.I.S Can Tho Hospital, statistical numbers showed an increasing of the overall neuro-interventional procedures 88.6% (891–1162), on average 74 (2019) to 96 (2020) cases per month, in comparison with last year. In the same period, rising of MT volumes and other interventional neuroradiology (INR) procedures was seen, 140% (45–108) and 84.6% (571–1054), respectively. Even during the 3 months (Apr–Jun) of Vietnamese nationwide lockdown, stroke care services were not being diminished but grown; an increasing of total neuro-interventional procedures and MT volumes; 85.8% (218–405) and 117.6% (17–37) compared with previous year. Many factors contributed to the stable development of stroke care in Mekong Delta, early admissions and pre-hospital recognition and improvement of stroke. More than that, geographical factors also contribute to the improvement of local stroke care, S.I.S Can Tho Hospital stand in the centre of Mekong Delta, covering for 17 million people, allowing most patients access to transfer in less than 2 h by ground transportation. Readiness of healthcare providers and the available medical facility – are consequences of effective public control, take key roles in improvement of stroke care, even during this COVID pandemic period.

## Neurological imaging and manifestations in COVID-19 patients

Acute stroke is the most common neuroimaging finding among hospitalized COVID-19 patients and its presence is a strong prognostic marker of poor outcome.^8^ A retrospective study of data from the COVID-19 outbreak in Wuhan, China, showed that the incidence of stroke among hospitalized patients with COVID-19 was ∼5%.^9^ COVID-19 infection had a significant independent association with acute ischemic stroke compared with control subjects in an American study. Of patients with acute ischemic stroke, 46.3% had COVID-19 infection compared with 18.3% of controls.^10^ Also patients with COVID-19 with a history of stroke had more severe clinical symptoms and poorer outcomes compared with those without a history of stroke.^11^ COVID-19 patients might present various central nervous system manifestations. Among 214 COVID-19 patients in a Chinese study, more than one-third of them had nervous system manifestations with the most common to be dizziness (16.8%), headache (13.1%), taste impairment (5.6%) and smell impairment (5.1%).^12^ Altered mental status was the second most common presentation in a UK study, comprising encephalopathy or encephalitis and primary psychiatric diagnoses, often occurring in younger patients.^13^

Precautions must be taken in neuroradiological departments when performing scans to protect patients, technologists and radiologists from infection. The organizational measures to cope with the influx of patients, while continuing to manage other emergency and time-sensitive activity have been discussed elsewhere.^14^ Key measures include dedicating a specific area to the evaluation of patients with suspected or confirmed COVID-19, keeping the attending staff to a minimum, triaging non-urgent procedures such as systematic evaluation of chronic diseases and screening procedure and giving highly frequent updates on the situation to the staff.

## General recommendations for INR activity

In any case scenario of the COVID-19 pandemic, the availability of an INR service for large vessel occlusion stroke and other emergencies had to be maintained. At the same time the resources of the healthcare systems had to be preserved for an eventual heavy strain due to large numbers of patients suffering from COVID-19 symptoms. Both goals seemed to have been achieved largely by achieving a high number of centres providing the usual emergency service combined with a profound reduction in elective procedures.^4^

Most published recommendations were realized during the lockdown period and the pandemic peak. The Society of NeuroInterventional Surgery,^15^ the European Society of Minimally Invasive Neurological Therapy,^16^ and the Society of Vascular and Interventional Neurology^22^ published individual recommendations and guidance statements for the care of emergent neurointerventional patients in the setting of COVID-19. The common goals for these recommendations were: (1) Diagnosis of potential COVID-19 patients presenting with neurovascular conditions and pre-hospital triage, (2) organization of the resources, service network, intra-hospital pathways and after care, (3) protection of healthcare workers (HCW), training and tele-management and (4) management of angiosuite and INR materials.

With time elapsing from the beginning of pandemic, recommendations and management can be divided into: (a) active pandemic phase; with the highest overload on health system, (b) re-opening phase; rescheduling elective interventions and managing workload and exhausted medical teams, (c) maintenance and alert phase; establishing the ‘usual’ work force and activity while getting ready for second (new) wave of active virus peak.

*(1) Diagnosis of potential COVID-19 patients presenting with neurovascular conditions and pre-hospital triage*

It is important in emergency stroke patients to classify them, if possible, into low risk, high risk or COVID-19 positive patients to choose the adequate pathway and level of protection to be applied. It might be beneficial to add a computed tomography (CT) chest to CT protocols of initial imaging for suspected stroke patients to detect any potential signs of COVID-19 infection.

If this is not feasible, a chest X-ray in angiosuite is recommended. For other INR emergencies, it is important to test the patients adequately to establish their infection status. All high-risk patients must be treated as COVID-19 positive patients and follow the COVID-19 designated pathways.^15,16^ Establishment of local coordination centres is encouraged and could facilitate routing of confirmed or suspected COVID-19 positive stroke patients to a hospital with the required critical care facilities. Direct transfer to a comprehensive stroke centre (CSC) would likely ensure the necessary expertise and resources in most cases, given that the majority of CSCs are large tertiary care centres.^17^ Alternatively, tele stroke systems can facilitate patient and neuroradiological assessment between PSC/acute stroke ready hospitals (ASRH) and CSCs, such that the CSC team can provide remote guidance for initial stroke management to the local PSC/ASRH team if necessary.^18^

*(2) Organize the resources, service network, intra-hospital pathways and after care*

Hospital resources are altered during the pandemic era and a thorough daily update about essential resources (available team members, intensive care beds, anaesthesiologists) will ensure a flawless workflow. Organizing the service network and patients’ pathway is crucial to decrease contamination, protect patients and medical staff and to provide a rapid management. All non-urgent INR procedures must be suspended, and patients’ consultations can be transformed into virtual ones.

Hospital capacity and adequate resources must be verified before initiating a transfer of any patent. Intra-hospital pathways for COVID-19 and high-risk patients must be well demarked and organized to ensure the least cross-over with other non-COVID patients and any medical staff not involved in patients care. It is recommended to the group all/most of medical management procedures (intubation, extubation and urethral catheter) into one place in a negative pressure room and to prophylactically intubate unstable patients before transfer to angiosuite.^15^ For COVID-free patients, transfer can be made to their hospital of origin to decrease the load on tertiary centres which are commonly a COVID-19 establishment.

*(3) Protection of HCW, training and potential tele-management*

COVID-19 is highly contagious and the potential risk for contamination among HCW is extremely high. In an early case series from Wuhan, China, 29% of patients with SARS-CoV-2 were healthcare workers and were assumed to have acquired the infection in the hospital.^19^

To protect the healthcare force, adequate use and periodic training on personal protective equipment (PPE) is mandatory. Mastering the strict rules and techniques of donning and doffing protective materials is essential. Decreasing direct contact inside healthcare facilities will decrease spreading of the disease for both HCW and patients. Measures for achieving this might include tele-meetings and tele-consultations for essential activity, limiting access and presence of only essential staff while managing patients, dividing the staff into two separate non-crossing teams and limiting staff presence at hospital for only essential medical acts. In a recent international survey, nearly half of participants reported to be working from their home office or are alternating between home office and the hospital, typically for a week at a time.^4^

*(4) Management of angiosuite and INR materials*

The angiosuite is considered a source of infection for both patients and HCW. It is advised to designate, if possible, a separate angiosuite for COVID-19 patients which will help in decreasing the risk of infection and will ensure a faster workflow for non-COVID-19 patients. Only essential materials for each procedure are to be present and strict respect of local disinfecting protocols is to be applied. It is also recommended to check periodically the negative air pressure environment and high efficiency particulate air filters of the angiosuites if present. The pandemic has also placed a strain on the fabrication of products, in addition to the delivery networks of industrial partners and it is recommended to ensure the availability of essential INR materials, that vital communications be established to share resources and information with other affiliated INR centres.^15,16^

## Remote solutions

The pandemic has led to a marked shift to remote solutions in the field with a possible positive impact in the future on hospital resources and medical workload. One major result of the COVID-19 pandemic and the need for social distancing is the rapid adoption of remote communication, particularly using conferencing tools. In a global survey, 33% of participants from all societies were interested in having access to 24/7 remote physician support through a live streaming platform.^4^ The interest in this technology ranged from 24% in Asia to 51% in Latin America. A recent study confirmed the feasibility of remote mentoring of thrombectomy procedures and the first small cases series provides evidence for the feasibility in an INR environment.^20^ Bechstein et al.^21^ reported the first clinical application of remote proctoring in the neurointerventional setting using a live-stream system specifically developed for this purpose. On-site presence of a proctoring specialist was not possible because of the COVID-19 pandemic. This technology could prove instrumental in including senior advice and guidance in emergency situations. This may remain critical in the future, as travel options may remain limited for an extended period of time.

## Aftermath of outbreak and second wave

It was a challenge on different levels to re-open centres and resume the elective activity in INR for different reasons; drained hospital resources and exhausted HCW, rescheduling all postponed and new patients, limited availability of staff to conduct multidisciplinary decision-making meetings and the fear of patients to be admitted in hospitals for elective interventions. The COVID-19 outbreak has also markedly changed the medical management of patients, training, and education. The fear of second wave is a reality with the advantage this time that teams are somewhat more familiar with the disease and relatively ready with protective and organizational protocols.

## Conclusion

Neurointerventional emergency services were available in almost all centres, while the number of emergency patients is markedly decreased. Many centres had abandoned neurointerventions in non-emergent situations. COVID19 created a dynamic situation with considerable variations in the management of neurointerventions and in the expectations for the future.
